# Prediction of protein–protein interaction sites by means of ensemble learning and weighted feature descriptor

**DOI:** 10.1186/s40709-016-0046-7

**Published:** 2016-07-04

**Authors:** Xiuquan Du, Shiwei Sun, Changlin Hu, Xinrui Li, Junfeng Xia

**Affiliations:** School of Computer Science and Technology, Anhui University, Hefei, 230601 Anhui China; Co-Innovation Center for Information Supply & Assurance Technology, Anhui University, Hefei, 230601 Anhui China; Key Laboratory of Intelligent Computing and Signal Processing of Ministry of Education, Anhui University, Hefei, 230601 Anhui China; Institute of Health Sciences, Anhui University, Hefei, 230601 Anhui China

## Abstract

**Background:**

Reliable prediction of protein–protein interaction sites is an important goal in the field of bioinformatics. Many computational methods have been explored for the large-scale prediction of protein–protein interaction sites based on various data types, including protein sequence, structural and genomic data. Although much progress has been achieved in recent years, the problem has not yet been satisfactorily solved.

**Results:**

In this work, we presented an efficient approach that uses ensemble learning algorithm with weighted feature descriptor (EL-WFD) to predict protein–protein interaction sites. Moreover, weighted feature descriptor was designed to describe the distance influence of neighboring residues on interaction sites. The results on two dataset (Hetero and Homo), show that the proposed method yields a satisfactory accuracy with 83.8 % recall and 96.3 % precision on the Hetero dataset and 84.2 % recall and 96.3 % precision on the Homo dataset, respectively. In both datasets, our method tend to obtain high Mathews correlation coefficient compared with state-of-the-art technique random forest method.

**Conclusions:**

The experimental results show that the EL-WFD method is quite effective in predicting protein–protein interaction sites. The novel weighted feature descriptor was proved to be promising in discovering interaction sites. Overall, the proposed method can be considered as a new powerful tool for predicting protein–protein interaction sites with excellence performance.

## Background

Protein–protein interactions (PPIs) are central to all aspects of biological systems including, for example, gene regulation, immunological recognition and protein synthesis [1, 2]. Exploiting the mechanisms of protein interactions plays a pivotal role for understanding the functions of biological systems. Hence, identification of binding sites between two interacting proteins is one of basic problems in the research of protein functions. Knowledge of the three-dimensional (3D) structure of the protein complex provides much valuable information on the protein interaction site. Several experimental technologies such as X-ray crystallography and NMR can be used to obtain such information. However, they cannot meet the requirements of proteomics-generated interaction data since they are time consuming and expensive. Therefore, reliable and efficient computational methods are required to assist the identification of protein–protein interaction sites.

A number of computational methods have been proposed for the prediction of interaction sites in proteins based on the sequence information [3, 4], 3D structure information [5] or a combination of 3D structure and sequence information. Machine learning methods such as support vector machine (SVM) [6–8], neural networks (NN) [9–12], Bayesian networks (BN) [13–16], random forests (RF) [17, 18], conditional random fields (CRF) [19], extreme learning machine (ELM) [20] and L1-logreg classifier [21] have been successful applied for predicting binding sites. Therefore, development of a machine learning based model using protein properties might be a promising strategy to predict unknown PPI sites.

In this study, we present a novel method for PPI sites discovery and prediction that uses weighted feature descriptor derived from protein sequence with ensemble learning algorithm. Firstly, we systematically investigated a wide variety of features from a combination of protein sequence and structure information, and then weighted feature descriptor (WFD) was used to encode the PPI sites. Secondly, meta-algorithm was chosen as the ensemble learning method to identify PPI sites. Finally, a new ensemble classifier, namely EL-WFD, was developed to further improve the prediction accuracy. To demonstrate its effectiveness, the proposed method was applied to both the Hetero and Homo datasets. Empirical studies showed the efficiency and effectiveness of our proposed approach.

## Results and discussion

### Comparing the prediction performance with/without WFD on the TRS

Four models were generated with/without WFD on the TRS, namely WFD-Hetero, noWFD-Hetero, WFD-Homo and noWFD-Homo, respectively. Then, fivefolds cross validation was used to evaluate the performance of different methods on the TRS. Table 1 shows the detail results of four methods. From Table 1, we can deduce that the average performance of WFD-Hetero is higher about 0.7 % in Accuracy, 0.4 % in Recall, 1.6 % in Precision than the noWFD-Hetero, respectively. The average performance of WFD-Homo is higher about 0.26 % in Accuracy and 0.9 % in Precision than the noWFD-Homo, respectively.

**Table 1:** The performance of four methods on the Hetero/Homo TRS

Method	Acc	Pre	Rec
noWFD-hetero	92.52	95.1	83.3
WFD-hetero	93.2	96.7	83.7
noWFD-homo	92.92	95.3	82.8
WFD-homo	93.18	96.2	82.8

*Acc* Accuracy, *Pre* Precision, *Rec* Recall

### Comparison with the other methods on the TES

In this study, we compared EL-WFD to J48 algorithm and RF using the same set. The results are shown in Table 2. The overall performance (Accuracy, Recall, Precision, F-measure and MCC) of our method were 93.11, 83.83, 96.3, 89.63 % and 0.8497 on the Hetero dataset. The success rate of J48 and RF was 88.29 and 76.15 % on the Hetero dataset. On the Homo dataset, the success rate of EL-WFD was 93.99 %. Hence, the success rate was improved by at least 6 %, while the overall Recall, Precision, F-measure and MCC were improved by at least about 1, 12, 6 and 10 % respectively.

**Table 2:** The performance comparison using different machine learning methods

Dataset	Classifier	Acc	Rec	Pre	F	MCC
Hetero	J48	88.29	83.29	83.69	83.49	0.7441
	RF	76.15	43.25	80.7	56.32	0.4576
	EL-WFD	93.11	83.83	96.3	89.63	0.8497
Homo	J48	87.97	81.51	80.62	81.06	0.7224
	RF	79.46	82.42	44.46	57.76	0.4956
	EL-WFD	93.99	84.19	96.34	89.86	0.8601

*Acc* Accuracy, *Rec* Recall, *Pre* Precision, *F* F-measure, *MCC* Mathews correlation coefficient

**Fig. 1:**
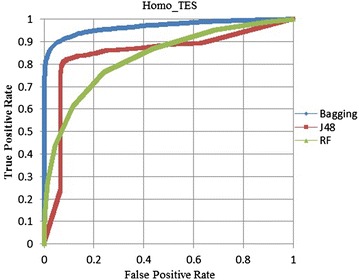
ROC curves on the homo TES

**Fig. 2:**
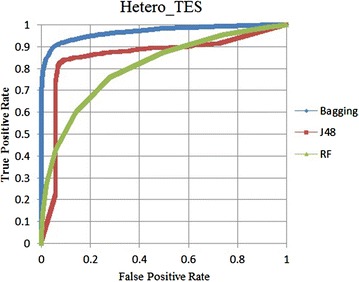
ROC curves on the hetero TES

ROC curves (Fig. 1) are also plotted to compare these three methods objectively on the Homo TES. From Fig. 1, it is found that Bagging is higher than the other methods on Homo test dataset. Figure 2 shows the same result with Fig. 1.

## Conclusions

In this paper, we have developed a new approach for PPI sites prediction, which combine ensemble learning method and weighted feature descriptor (EL-WFD). EL offers significant advantages such as fast learning speed, ease of implementation, better generalization performance, and least human intervention. WFD is an effective feature representation method, which can uncover distance influence of neighboring residues on interacting sites. Experimental results show that our method performed significantly well in distinguishing interacting and non-interacting sites. In both datasets, our method tend to obtain high Mathews correlation coefficient (MCC) compared with state-of-the-art technique random forest method. In the future, we will focus on how to predict hot spots in protein interfaces.

## Methods

### Generation of the datasets

We evaluated the proposed method with the same dataset used in the study of Koike et al. [22]. The PPI sites dataset was collected from the Protein Data Bank (PDB). The protein pairs which contain a protein with few than 100 residues, or have more than 25 % sequence identity were removed. In addition, the number of protein–protein interaction sites less than 20 interfacial residues and 30 interfacial residues for heterocomplex and homocomplexes respectively, or no HSSP entry available [23]are removed. The remaining 559 non-redundant chains, where 270 are from hetero complexes and the other 289 are from homo complexes, comprise the final dataset. We randomly select 202 chains of all chains as Train Set (TRS) and 87 chains as Test Set (TES) from Homo dataset. We also select 189 chains of all chains as TRS and 81 chains as TES from Hetero dataset.

### Definition of protein interaction sites

Interfaces are formed mostly by residues that are exposed to the solvent if the partner chain is removed, so we focus on surface residues for later prediction. A residue is considered to be a surface residue if at least 16 % of the solvent accessible surface area (ASA) was exposed to solvent [24]. The ASA of each residue in the unbound molecule (MASA) and in the complex (CASA) is computed using the DSSP program [25]. Meanwhile, a surface residue is defined to be an interface residue if it formed an interfacial contact (|MASA-CASA| >=1).

### Feature extraction for residues

To use machine learning methods to predict PPI sites, one of the most important computational challenges is to extract the biological characteristics in which the important information content of amino acid residues is fully encoded. In this study, we extracted feature vectors based residue structure, sequence, and physicochemical information.

#### Structure based features

1. Accessible surface area: The accessible surface area (ASA) is the atomic surface area exposed to a solvent. The ASA value of each residue calculated by DSSP was used in our work. In addition, protein structure and interaction analyzer (PSAIA) calculates the ASA value for each residue, including backbone ASA, side-chain ASA, polar ASA and non-polar ASA.
2. Relative accessible surface area: Relative accessible surface area (RASA) extracted in this work was calculated by PSAIA [26]. The following residue attributes are calculated by PSAIA: total RASA, backbone RASA, side-chain RASA, polar RASA and non-polar RASA.
3. Depth index: The residue depth is defined as the minimum distance of a residue from any solvent accessible residue and it has been computed by PSAIA. For residue depth, there are six features were calculated by PSAIA. In this paper, the average depth index (DPX) is used.
4. Protrusion index: The protrusion of a non-hydrogen residue is the ration of the volume of a sphere with a radius of 10.0 Å centered at that atom that is not filled with atoms. Same with the DPX, PSAIA calculates six features for the protrusion and the average protrusion index (CX) is adopted.

#### Sequence based features

1. Properties from HSSP file: The sequence profile in HSSP file for each protein chain are composed of L rows and 20 columns. ‘L’ stands for the number of amino acids in a chain and 20 kinds of amino acids index columns. P_i,j_ means the probability of j-th amino acid take the place of the i-th residue. We also extracted the other four properties of protein from HSSP [27] database: entropy, relative entropy, conservation weight and sequence variability. Entropy measures the conservation of a residue in the location. Relative Entropy is defined as the standardized entropy which normalized to the scale of 0–100. Conservation Weight measures the sequence conservation of a position. Sequence variability contains evolutionary information, on a scale of 0–100 as exported from NAGLIN alignments.

#### Physicochemical features

1. High-quality-indices: Saha et al. [28] have made a conclusion that physicochemical features of amino acids play a significant role in identifying the PPI sites, thus properties of amino acids are taken into count as important characteristics in discriminating between interacting sites and non-interacting sites. Recently, 544 physicochemical and biochemical properties of amino acids are released in AAIndex1 database. Based on the statistical analyses, Saha et al. [28] categorized these 544 characteristics into eight classes, named high-quality-indices (HQIs). In this work, the HQI8 containing eight clusters named electric properties (BLAM930101), hydrophobicity (BIOV880101), alpha and turn propensities (MAXF760101), physicochemical properties (TSAJ990101), residue propensity (NAKH920108), composition (CEDJ970104), beta propensity (LIFS790101) and intrinsic propensities (MIYS990104), respectively. Each cluster is composed of one value and there are eight indices for each amino acid.
2. Amino acid factors: Based on AAindex1, Atchley et al. [29] made statistical analyses on these 544 properties, as well. Different from HQI, they summarized these properties into five patterns, which reflect polarity, secondary structure, molecular volume, codon diversity and electrostatic charge. These features were also used to evaluate protein interaction sites.

### The WFD of the residue

The environment factors for each residue position are very important for PPI sites, so the profiles of sequentially neighboring residues or spatially neighboring residues were adopted as residue features in PPI site prediction in previous report [20]. However, distance effect among these sequentially neighboring residues or spatially neighboring residues was not considered. In other words, as the distance between the query residue and its neighboring residue increases, the neighboring residue will have smaller effect on the query residue, and vice versa. Therefore, we propose a novel WFD which considers the distance effect among the query residue and its neighboring residue. To illustrate the WFD, for example, given the protein sequence segment SLDIQSAA and Q is the interaction site (query residue). In this case, the sliding window is fixed to five and sequentially neighboring is considered. Thus, the feature vector components were arranged in ascending order according to the distance between the neighboring residues, which can be defined as follows,

 ($$V = V_{D} ,V_{I} ,V_{Q} ,V_{S} ,V_{A}$$),

where $$V_{residue} = (HSSP,PSAIA,HIQ,AAFactors),residue = D,I,Q,S,A .$$

Second, we calculate the distance effect according to $C_{\alpha }$-atom coordinate of D, I, Q, S and A. Here, the Euclidean distance is used to evaluate the distance effect among the residue, which can be calculated as,

$${\text{ED}}_{\text{D,Q}} = \sqrt {(x_{D} - x_{Q} )^{2} + (y_{D} - y_{Q} )^{2} + (z_{D} - z_{Q} )^{2} }$$

$${\text{ED}}_{\text{I,Q}} = \sqrt {(x_{I} - x_{Q} )^{2} + (y_{I} - y_{Q} )^{2} + (z_{I} - z_{Q} )^{2} }$$

$$ED_{Q,Q} = 1$$

$${\text{ED}}_{\text{S,Q}} = \sqrt {(x_{S} - x_{Q} )^{2} + (y_{S} - y_{Q} )^{2} + (z_{S} - z_{Q} )^{2} }$$

$${\text{ED}}_{\text{A,Q}} = \sqrt {(x_{A} - x_{Q} )^{2} + (y_{A} - y_{Q} )^{2} + (z_{A} - z_{Q} )^{2} }$$

where $x_{D}$ denotes the x coordinate of $C_{\alpha }$-atom, $y_{D}$ denotes the y coordinate of $C_{\alpha }$-atom, and $z_{D}$ denotes the z coordinate of $C_{\alpha }$-atom of residue D, respectively. The rest symbols have similar meanings as those used for residue D.

Finally, the WFD can be written as,

$$WFD_{Q} = (\frac{{V_{D} }}{{ED_{D,Q} }},\frac{{V_{I} }}{{ED_{I,Q} }},\frac{{V_{Q} }}{{ED_{Q,Q} }},\frac{{V_{S} }}{{ED_{S,Q} }},\frac{{V_{A} }}{{ED_{A,Q} }}) = (\frac{{V_{D} }}{{ED_{D,Q} }},\frac{{V_{I} }}{{ED_{I,Q} }},V_{Q} ,\frac{{V_{S} }}{{ED_{S,Q} }},\frac{{V_{A} }}{{ED_{A,Q} }})$$

### The feature space

For each residue, 50 features were extracted including 25 features from HSSP profile, 12 features from structure information (5 features from ASA, 5 features from RASA, 1 feature from DPX, 1 feature from CX), and 13 features from physicochemical information (8 features from HQI8 and 5 features from amino acid factors). In addition, taking into consideration the effect of neighbor residues, 11-size sliding window is used to describe current residue. Therefore, 50×11 = 550 features were extracted for each residue.

### Ensemble meta-algorithm

In this paper, bagging algorithm is used to implement ensemble meta-algorithm which improves the stability and accuracy of machine learning algorithms used in statistical classification and regression. It also reduces variance and avoids over-fitting. Although it is usually applied to decision tree methods, it can be used with any type of method.

Suppose a standard training set $D$ of size $n$, bagging will produce $m$ new training sets $D_{i}$ with size $n'$, by sampling from D uniformly and with replacement. By sampling with replacement, some observations may be repeated in each $D_{i}$. If $n\prime = n$, then for large $n$ the set $D_{i}$ is expected to have the fraction (1−1/e) (≈63.2 %) of the unique examples of D, the rest being duplicates. This kind of sample is known as a bootstrap sample. The $m$ models are fitted using the above $m$ bootstrap samples and combined by voting for classification.

### Performance evaluation

PPI sites prediction is a binary classification problem. In this experiment, precision (Pre), recall (Rec), accuracy (Acc), F-measure (F), and Matthews correlation coefficient (MCC) were employed to measure the performance of classifiers:

1 $${\text{Re}}c\;{ = }\frac{TP}{TP + FN}$$

2 $$\Pr e = \frac{TP}{{TP\text{ + }FP}}$$

3 $$Acc = \frac{TP + TN}{TP + TN + FN + FP}$$

4 $$F = \frac{{2 \times \Pr e \times \text{Re} c}}{{\Pr e + \text{Re} c}}$$

5 $$MCC = \frac{TP \times TN - FP \times FN}{{\sqrt {(TP + FP) \times (TP + FN) \times (TN + FP) \times (TN + FN)} }}$$

where true positive (TP) denotes the number of true interaction site, true negative (TN) denotes the number of true non-interaction site, FP (False Positive) denotes the number of false interaction site, and false negative (FN) denotes the number of false non-interaction site. The ROC curve is often used to evaluate classifier performance. A classifier conducts predictions on the basis of a threshold, which generally is defined as 0.5. When the threshold value is changed, new predictions can be obtained and a point can be plotted with the true positive rate (TPR) *versus* the false positive rate (FPR) for different threshold values.

6 $$TPR = \frac{TP}{TP + FN}$$

7 $$FPR = \frac{FP}{TN + FP}$$

The area under a curve (AUC) for the receiver operating characteristic (ROC) curve is also used. When the AUC value of a predictor is larger than the area of other ROC curves, such a predictor is considered better than other predictors.
